# Intravitreal ziv-aflibercept in diabetic vitreous hemorrhage

**DOI:** 10.1186/s40942-019-0204-9

**Published:** 2020-01-14

**Authors:** Ahmad M. Mansour, Mohammed Ashraf, Khalil M. El Jawhari, Michel Farah, Ahmed Souka, Chintan Sarvaiya, Sumit Randhir Singh, Alay Banker, Jay Chhablani

**Affiliations:** 1 Department of Ophthalmology, American University of Beirut, Beirut, Lebanon; 2 Department of Ophthalmology, Rafic Hariri University Hospital, Beirut, Lebanon; 3 Department of Ophthalmology, Alexandria Faculty of Medicine, Alexandria University, Alexandria, Egypt; 4 Medical University of Lodz, Lodz, Poland; 5 Department of Ophthalmology, Federal University of São Paulo, São Paulo, Brazil; 6 Banker Retina Clinic and Laser Center, Ahmedabad, Gujarat India; 7 Smt Kanuri Santhamma Centre for Vitreoretinal Diseases, LV Prasad Eye Institute, Hyderabad, Andhra Pradesh India

**Keywords:** Intravitreal ziv-aflibercept, Panretinal photocoagulation, Proliferative diabetic retinopathy, Vascular endothelial growth factor, Vitreous hemorrhage

## Abstract

**Background:**

To evaluate the safety and efficacy of intravitreal ziv-aflibercept (IVZ) in the management of vitreous hemorrhage (VH) in eyes with previously lasered proliferative diabetic retinopathy (PDR).

**Methods:**

In a prospective multicenter study, previously lasered eyes who had dense VH from PDR underwent intravitreal injection of ziv-aflibercept (IVZ) (1.25 mg aflibercept). Demographic characteristics of the patients, baseline and final logMar visual acuity, number of injections, VH clearance time, and need for vitrectomy were recorded.

**Results:**

Twenty-seven eyes of 21 patients were included in the study. Mean age of study patients was 61.3 ± 14.1 years with mean duration of diabetes mellitus of 22.6 ± 7.8 years. Mean logMAR BCVA at baseline was 1.41 ± 1.26 (Snellen equivalent 20/514) and at the last visit 0.55 ± 0.61 (Snellen equivalent 20/70) with a mean gain of 0.86 EDTRS line (paired student *t* test = 5.1; p ≤ 0.001). Mean number of IVZ 2.4 ± 1.6 (range 1–6). The mean follow-up time was 11.7 ± 11.1 months (range 1–34). Mean time for visual recovery and/or VH clearance was 5.7 ± 3.3 weeks. Eyes, which required multiple injections, the interval period between injections for recurrent VH was 6.4 ± 5.2 months. No subject required vitrectomy. No ocular or systemic adverse effects were noted.

**Conclusions:**

IVZ injections had good short-term safety and efficacy for the therapy of new or recurrent VH in previously lasered eyes with PDR reducing somewhat the need for vitrectomy.

*Trial registration*: NCT02486484

## Introduction

Panretinal photocoagulation (PRP) is still one of the gold standards for treating proliferative diabetic retinopathy (PDR), yet many patients have an incomplete response with subsequent risk of vitreous hemorrhage (VH) and vision loss [1–5]. Spaide and Fisher [1] pioneered the use of vascular endothelial growth factor (VEGF) antagonists in eyes with PDR and VH. Several clinical trials [4–12] have provided evidence that intravitreally administered anti-VEGF drugs (bevacizumab or ranibizumab) may induce a short-term regression of new vessels in PDR with or without VH. Ziv-aflibercept (Sanofi and Regeneron Pharmaceuticals, Inc. Tarrytown, NY, USA) is the off-label equivalent to the approved aflibercept (Eylea, Regeneron, Tarrytown, NY, USA) for the treatment of neovascular age-related macular degeneration, and diabetic macular edema (DME). In this study, we evaluated the effect of intravitreal ziv-aflibercept in eyes with VH and PDR following full PRP.

## Methods

This prospective uncontrolled clinical study included 27 eyes of 21 patients from January 2015 to August 2018. This study was performed at 3 sites (Lebanon, Egypt and India). The study and data accumulation were carried out with approval from the Institutional Review Boards at each site (registered as NCT 02486484). This study adhered to the tenets of the Declaration of Helsinki. All participants were informed in detail about the nature of off-label use of this medication and the possible risks. Informed consent was obtained from each participant. Eligible patients with dense VH secondary to PDR with visual acuity equal or worse than 20/80 were enrolled in the study. PDR eyes in our study had persistent active retinal neovascularization despite prior complete PRP (confluent laser marks covering from equator to the retinal periphery with a minimum of 1200 laser spots). Inclusion criteria were age of 18 years or older with type 1 or 2 diabetes mellitus, PDR (neovascularization at the optic disc or elsewhere) with recurrent fresh dense VH, prior PRP of more than 2 months duration. Dense VH was defined as obscuration of posterior pole details and visual drop to 20/80 or worse. The exclusion criteria were as follows: patients with a single eye, history of glaucoma, ocular inflammation, and tractional retinal detachment (by B-scan or prior history). Ziv-aflibercept 0.05 mL (1.25 mg aflibercept) was prepared and injected according to standard protocols (compounding under sterile conditions with storage at 4 °C for 4 weeks). The intravitreal injection was performed under sterile conditions and using topical anesthetic 3.5 mm from the limbus via a 30-gauge needle. Best-corrected visual acuity (BCVA) was taken with a Snellen acuity chart and converted to logarithm of minimum angle of resolution (logMAR) equivalent units for statistical calculations. VH clearance time was defined as the time until vessels in the posterior pole were clearly visible. Recurrent VH was defined as fresh hemorrhage developing after resolution of previous hemorrhage or worsening of current hemorrhage. The patients were followed monthly till clearance of the vitreous hemorrhage. Thereafter patients were instructed to return at the first sign of acute drop in vision, with the instruction to check the vision in each eye separately.

## Results

A total of 21 consecutive patients (27 eyes-13 right and 14 left) with PDR previously lasered and dense VH received at least 1 IVZ injection. VH was of acute onset (less than 2 weeks) except in one case with chronic VH of 3 months duration. Patient distribution included 11 males and 10 females, 20 Caucasians, and 1 Asian Indian. The mean age of patients was 61.3 ± 14.1 years (range: 31–88 years). The mean duration of diabetes was 22.6 ± 7.8 years (range: 6–40 years). Demographic data of all patients are presented in Table 1. There was a statistically significant change in mean BCVA from 20/514 (1.41 ± 1.26 logMAR) at baseline to 20/70 (0.55 ± 0.61 logMAR) at last follow-up visit (paired student t = 4.74; p < 0.001). Mean number of injections was 2.4 ± 1.6 (range: 1–6). Mean follow-up period was 11.7 ± 11.1 months (range: 1–34 months). Mean interval between repeated injections was 6.4 ± 5.2 months (range: 1–27 months). Mean time for visual recovery and/or VH clearance was 5.7 ± 3.3 weeks (range: 2–12 weeks). Reinjection was required in 17 eyes because of recurrence of VH in 16 eyes and non-clearing VH in 1 eye. No serious ocular or systemic side-effects were detected.

**Table 1:** Clinical characteristics of 21 patients who received intravitreal ziv-aflibercept injections for dense vitreous hemorrhage secondary to refractory proliferative diabetic retinopathy with previous panretinal photocoagulation

Number	Age	Gender	OD or OS	Initial vision	Final vision	Number of injections	Follow-up (months)	Interval between injections (months)	Mean time for recovery (weeks)
1	75	F	OS	20/200	20/200	4	9	5;1;2	6
2	65	M	OD	HM	20/400	1	3	–	4
3	31	F	OD	20/400	20/80	1	2	–	4
4	31	F	OS	20/125	20/125	1	3	–	8
5	65	F	OS	20/125	20/50	2	6	1	12
6	60	F	OS	20/200	20/200	1	2	–	4
7	52	F	OD	20/100	20/30	2	4	1	12
8	55	M	OD	20/2000	20/20	1	2	–	4
9	51	M	OS	CF2m	20/300	1	1	–	2
10	64	F	OS	CF2m	20/100	2	27	27	2
11	56	M	OD	CF4m	20/50	3	3	1	4
12	52	M	OD	HM	20/30	6	34	10;3;3;10;6	6
13	52	M	OS	CF3m	20/60	5	32	10;7;10;6	8
14	57	M	OS	20/80	20/50	5	18	1;3;5;10	2
15	57	M	OD	20/100	20/50	2	8	2	6
16	64	M	OD	20/100	20/50	1	8	–	1
17	33	F	OD	HM	20/150	5	33	12;3;12;12;7	8
18	33	F	OS	20/200	20/50	1	12	–	NA
19	74	M	OS	CF1m	20/50	3	20	7;11	2
20	73	M	OD	CF2m	20/25	2	3	3	5
21	73	M	OS	HM	20/40	2	2	1.3	7
22	66	F	OD	20/200	20/60	1	19	–	8
23	66	F	OS	CF1m	CF3m	5	30	8;6;7;9	10
24	84	M	OS	HM	CF3m	2	12	11	2
25	88	M	OD	HM	CF5m	2	17	1	4
26	55	F	OD	20/200	20/140	1	3	–	12
27	68	F	OS	CF1m	20/80	2	9	9	6

## Discussion

The natural history of diabetic VH without prior PRP is poor. In a study of 85 eyes followed 3–10 years for VH, 25 eyes (30%) improved while 68% ended up with a visual acuity of 5/200 or worse [13]. PRP is a mainstay treatment for PDR due to the long-term suppressive effect on retinal neovascularization [3]. In a meta-analysis, PRP reduced each of the following by around 50%: risk of progression of PDR, risk of new VH, and risk of vision loss 3. In about a third of cases, retinal new vessels either continue to grow or do not regress despite full PRP leading to recurrent VH [14, 15].

The natural history of diabetic VH in eyes with previous PRP is still undefined in the literature but appears more favorable than in eyes without PRP [4]. In these situations, intravitreal VEGF antagonists can hasten visual recovery in a majority of cases [6], while vitrectomy is reserved for recalcitrant cases [16]. Sinawat et al. [6] evaluated the efficacy of intravitreal bevacizumab in 18 previously lasered eyes with new dense VH: VH cleared completely in 7 (38.8%), 9 (50%), and 13 (72.2%) eyes after 1.5, 6, and 12 months, respectively. Our results were more encouraging as visual recovery occurred within several weeks rather than several months. Earlier intervention in the setting of fresh VH, more extensive peripheral PRP and possibly different VEGF potency of the injected drug may explain the fast visual recovery with relatively rapid clearance of VH. It is important to note that recurrent VH is not prevented by vitrectomy but is also controlled by anti-VEGF injections. In a controlled study of post-vitrectomized PDR eyes with postoperative recurrent VH [12], vitrectomy was repeated in 8 of 18 controls and in none of 20 eyes that received intravitreal bevacizumab injections. VH can resolve spontaneously [13], but VEGF antagonists appear to speed up resolution by inducing vasoconstriction of the bleeding new vessel(s) and cessation of active bleeding. While early vitrectomy for dense VH can be sight saving when PDR is previously untreated [14], there is less concern when the eye had prior PRP [4].

It appears that no prior study has examined the efficacy of intravitreal aflibercept in diabetic VH with or without prior PRP, although such studies were previously carried with intravitreal bevacizumab or ranibizumab [6–12]. Though aflibercept and ziv-aflibercept have an identical molecular structure, the difference exists in the osmolarity (300 mOsm/kg for aflibercept vs. 1000 mOsm/kg for ziv-aflibercept) due to difference in purification and buffer use. It had been proposed that vitreous osmolarity would increase by only 4% (300 mOsm/L to 312 mOsm/L) in an emmetropic human eye (4.4 ml) after the injection of 1.25 mg ziv-aflibercept, which remains well below the 500 mOsm/L threshold required to damage the RPE [17]. In general, it appears that anti-VEGF therapy may be more cost effective for VH in eyes with lasered PDR than for diabetic macular edema because it achieves large visual gains with fewer injections. Specifically, early use of ziv-aflibercept can be cost-effective since the patient recovers vision within weeks. The anticipated medical costs of IVZ therapy include mainly the costs for professionals, hospital, and drugs (30 USD per dose). Based on cost savings, this option (IVZ) appears quite similar to intravitreal bevacizumab (60 USD per compounded dose), but far less expensive to either intravitreal ranibizumab or aflibercept (30 times the price of compounded IVZ). Early vitrectomy for diabetic VH was previously shown to be cost-effective [18], but IVZ appears much more cost-effective compared to surgical intervention (additional costs of 8706 USD for vitrectomy) [19].

The limitations of our study include the small sample size and the limited follow-up, not accounting for systemic factors such as uncontrolled diabetes (hemoglobin A1C level), systemic blood pressure, smoking status, obesity, history of chronic obstructive lung disease, presence of anemia, anticoagulant use, presence of cough, sleep apnea, etc. There is one pilot study that support the efficacy of intravitreal bevacizumab injection in eyes with VH and PDR with previous complete PRP. We add here the efficacy and safety of early IVZ in managing VH in PDR eyes that received prior PRP.

## Conclusion

IVZ injections had good short-term safety and efficacy for the therapy of new or recurrent VH in previously lasered eyes with PDR reducing somewhat the need for vitrectomy.

## Summary

### What was known before

- Little is known about the natural history of vitreous hemorrhage in eyes with lasered proliferative diabetic retinopathy.
- A single pilot study supported the efficacy of intravitreal bevacizumab in eyes with diabetic vitreous hemorrhage and previously lasered.

### What the study adds

- Intravitreal ziv-aflibercept injections allowed visual recovery within weeks in eyes with dense vitreous hemorrhage from proliferative diabetic retinopathy that previously received scatter panretinal photocoagulation.
- Intravitreal ziv-aflibercept injections are cost-effective in managing diabetic vitreous hemorrhage compared to vitrectomy or intravitreal ranibizumab or aflibercept.

## Data Availability

The datasets used and/or analysed during the current study are available from the corresponding author on reasonable request.
